# The effect of COVID-19 on orthopedic elective/emergency procedures in a tertiary hospital Riyadh Saudi Arabia. A cross-sectional study

**DOI:** 10.1016/j.amsu.2022.104331

**Published:** 2022-08-11

**Authors:** Abdulellah Abunayan, Bader Aljadaan, Mohammed Almudayfir, Sayaf Alshareef, Abdulaziz alamer

**Affiliations:** Department of Orthopedic, Prince Sultan Military Medical City, Riyadh, Saudi Arabia

**Keywords:** COVID-19, Orthopedic procedures, Elective procedure, Pandemics, Saudi Arabia

## Abstract

**Background:**

The occurrence of the COVID-19 pandemic in late December 2019 created a worldwide emergency. It affected most surgical subspecialties in many ways. Therefore, we aimed to quantitate the early effect of COVID-19 on elective and emergency orthopedic surgeries. Moreover, to identify the most affected orthopedic subspecialties during this crisis.

**Materials and methods:**

Our study was conducted in a tertiary hospital in Saudi Arabia. We included all patients who underwent orthopedic procedures from January 1, 2020 - June 30, 2020, with the same period of 2019 for comparison. Emergency procedures were considered if a patient had an acute fracture, joint dislocation, compartment syndrome, infection, and infected non-union.

**Results:**

The impact of procedure cancellation in the early COVID-19 phase was significant. Our department procedures decreased in the 2nd quarter of 2020 by 75.6% from the previous quarter of the same year and 61.1% from the 2nd quarter of 2019. No admission restrictions were made for oncology and trauma patients, though they had a reduction in their cases. The most affected subspecialties in our study were sports medicine, upper limb, arthroplasty, and pediatrics.

**Conclusion:**

During the COVID-19 crisis, elective surgeries were held in our hospital and most of Saudi Arabia. Our cohort showed a significant decrease during this period. All subspecialties were affected by elective procedure cancellation, but some were affected more because of the elective nature of their operations, such as sports and upper limb, pediatric, and arthroplasty.

## Highlights

• COVID-19 had created a significant emergency in healthcare systems worldwide.
• Most orthopedic procedures are elective.
• COVID-19 caused a significant decrease in all elective orthopedic procedures.
• We believe sports, upper limb, pediatrics, and arthroplasty were affected the most.

## 1 Introduction

The first occurrence of COVID-19 in China Wuhan district was in late December 2019 [1]. This led to a cascade of events when the World Health Organization (WHO) characterized COVID-19 as a pandemic [2]. The Saudi Ministry of Health (MOH) announced the first case in Saudi Arabia on March 2nd, 2020 [3]. In the beginning, some governments trusted their healthcare systems and attempted to avoid an economic crisis where they only implemented social distancing, which quickly shifted to a whole country lockdown owing to the catastrophic surge of cases and mortality like Italy [4]. On the other hand, some governments adopted the entire country lockdown from the beginning to avoid early healthcare system fatigue and resource depletion, such as Saudi Arabia [5]. The latter approach had disrupted the usual daily living, economy, and social gathering. Many healthcare systems, including ours, decided to cancel or reduce elective hospital admissions along with routine and unnecessary outpatient visits. The reasoning for such action was to allocate the workforce to areas in need, protect the vulnerable population from acquiring the virus in hospitals, utilize wards, intensive care unit (ICU) beds, ventilators to almost full capacity for COVID-19 infected patients, and preserve resources to fight the emerging virus [[6], [7], [8], [9], [10], [11], [12]]. As orthopedic surgeons at the beginning of this pandemic, we were not expected to deal directly with infected patients as our fellows who covered medical, emergency, isolation, and ICU services. But as the healthcare system was stretched to the maximum, orthopedic teams with other surgical specialties were called to help [6,10,12]. The catastrophic effect of this global pandemic on the economy, healthcare systems, countries' resources, and lives cannot be unnoticed, with 6,261,708 deaths worldwide registered on May 14th, 2022 [13]. This study quantitates the early effect of COVID-19 on elective and emergency orthopedic surgeries in our institute. Moreover, to have an idea of the most affected subspecialties to guide orthopedic departments in allocating resources and expanding intra-departmental plans.

## 2 Methods

**Fig. 1:**
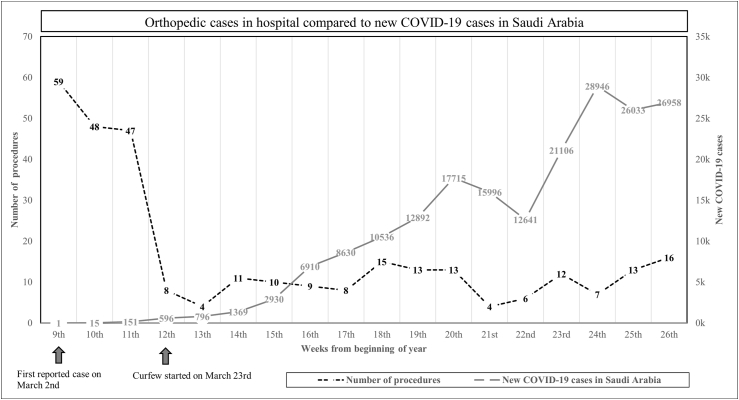
Orthopedic cases in hospital compared to new COVID-19 cases in Saudi Arabia.

A retrospective cross-sectional study design was used to measure the change in orthopedic procedures during the first half of 2020 compared to the same period of 2019. Our study was conducted in a tertiary hospital in Riyadh, Saudi Arabia. The study included all patients who underwent orthopedic procedures from January 1, 2019 to June 30, 2019 and January 1, 2020 to June 30, 2020. We chose this period because the first reported case of COVID-19 was March 2nd, 2020 (the 9th week from the beginning of the year), and the curfew started on March 23rd, 2020 (the 12th week from the beginning of the year). Therefore, the admission to our hospital was affected at the beginning of the curfew [Figure-1]. Moreover, we wanted to compare the most affected quarter (2nd quarter 2020) to the 1st quarter 2020 and the same quarter of the previous year (2nd quarter 2019). The effect of the curfew on admission is not our primary objective but to document the timeline of the drop in orthopedic procedures. Thus, we considered the 12th week as part of the 1st quarter of 2020. A non-randomized non-probable consecutive sampling was utilized on all orthopedic patients within the same period. Our department has six units: trauma, sports & upper limb, foot & ankle, pediatrics, oncology, and arthroplasty. While spine surgery is considered a different department, and hand surgery is covered by plastic service. Therefore, their cases were excluded. A procedure is counted if a patient entered the operation room for an orthopedic intervention either as a single or multiple simultaneous procedures (e.g., if a poly-trauma patient underwent multiple bone fixation in a single session, it was counted as one procedure). However, if a patient had multiple operations with different sessions, it was counted as multiple procedures (e.g., if a patient underwent multiple irrigation and debridement, it was registered as separate procedures). The urgency of a procedure was divided into elective or emergency. Elective procedures in our hospital included a variety of cases such as deformity correction, upper and lower limb joint arthroplasty, upper and lower limb arthroscopy, tendon transfer, tendon lengthening, open biopsy, mass excision, non-union, and malunion. On the other hand, emergency procedures included acute fractures, joint dislocation, compartment syndrome, infection, and infected non-union. The data was collected from our official theater logbook registry. All elective admissions were put on hold in our hospital before the curfew law. This included minimal invasive procedures as well as day surgeries. The researchers reviewed all patients from our medical records system. Data was gathered through a google form that was converted to Excel 2019 sheet. Our data consisted of patients' basic demographics, date of surgery, the urgency of the procedure, the subspecialty patient admitted under, and the number of consultants in each unit. Data about the COVID-19 cases were taken from the official MOH registry [14]. All data were calculated by SPSS (IBM Corp. Released 2012. IBM SPSS Statistics for Windows, Version 21.0. Armonk, NY: IBM Corp.). Continuous variables were expressed as mean ± standard deviation, and categorical variables were expressed as frequencies and percentages. We used the Chi-square test or Fisher's exact test to compare nominal variables. Moreover, we utilized an independent *t*-test to compare means for all continuous variables. A p-value <0.05 was considered statistically significant.

Our work has been reported in line with the STROCSS criteria [15]. This research was registered on www.researchregistry.com (registration number: researchregistry8037).

## 3 Results

### 3.1 First six months of 2019

Our study had 806 procedures in the first six months of 2019, of which 507 (62.9%) were males with a mean age of 35.02 (Standard deviation = 22.86) years. Of those procedures, 506 (62.8%) were elective procedures. We had 13 consultants covering our department units and a newly established foot & ankle (F&A) unit covered by one consultant. In the 1st quarter (Q), we performed a total of 454 procedures, of which 177 (39%) of them were emergency operations, and 289 (63.7%) were males with a mean age of 34.39 (Standard deviation = 22.09) years (Table 1). Most procedures were performed under the care of the trauma unit 129 (28.4%), followed by sports and upper limb 122 (26.9%), arthroplasty 83 (18.3), pediatrics 81 (17.8%), oncology 35 (7.7%) and foot and ankle unit with 4 (0.9%) cases. In the 2nd Q, we did 352 procedures in which 123 (34.9%) were emergency, and 218 (61.9%) were males, with a mean age of 35.85 (Standard deviation = 23.82) years (Table 1). The specific subspecialty distribution is shown in Table 2.

**Table 1:** Basic demographic characteristics by quarter.

Year	Quarter	Variable		N (*SD*/%)
**2019** **N = 806**	**1**st **Q**^a^**n = 454**	Age		34.39 ***(22.09)***
		Gender	Female	165 (36.3)
			Male	289 (63.7)
		Type of surgery	EL^b^	277 (61)
			EM^b^	177 (39)
	**2**nd **Q**^a^**n = 352**	Age		35.85 ***(23.82)***
		Gender	Female	134 (38.1)
			Male	218 (61.9)
		Type of surgery	EL^b^	229 (65.1)
			EM^b^	123 (34.9)
**2020** **N = 700**	**1**st **Q**^a^**n = 563**	Age		39.3 ***(22.37)***
		Gender	Female	229 (40.7)
			Male	334 (59.3)
		Type of surgery	EL^b^	388 (68.9)
			EM^b^	175 (31.1)
	**2**nd **Q**^a^**n = 137**	Age		37.03 ***(25.54)***
		Gender	Female	48 (35)
			Male	89 (65)
		Type of surgery	EL^b^	28 (20.4)
			EM^b^	109 (79.6)

a Quarter.

b Elective surgery (EL) and emergency surgery (EM).

**Table 2:** Comparison between 2nd Q^±^ 2020 and 2nd Q^±^ 2019 and 1st Q^±^ 2020.

Variable			2nd (Q^±^) 2019	2nd (Q^a^) 2020	P-value	1st (Q^a^) 2020	2nd (Q^±^) 2020	P-value
			n = 352 *(SD*/%)	n = 137 *(SD*/%)		n = 563 *(SD*/%*)*	n = 137 *(SD*/%*)*	
**Age**			35.85 ***(23.82)***	37.03 ***(25.54)***	0.63	39.3 ***(22.37)***	37.03 ***(25.54)***	0.34
**Gender**		Female	134 (38.1)	48 (35)	0.53	229 (40.7)	48 (35)	0.22
		Male	218 (61.9)	89 (65)		334 (59.3)	89 (65)	
**Type of surgery**		EL^b^	229 (65.1)	28 (20.4)	<0.001*	388 (68.9)	28 (20.4)	<0.001*
		EM^b^	123 (34.9)	109 (79.6)		175 (31.1)	109 (79.6)	
**Subspeciality**	Trauma	EL^b^	32 (36.8)	5 (10)	0.001*	49 (37.1)	5 (10)	<0.001*
		EM^b^	55 (63.2)	45 (90)		83 (62.9)	45 (90)	
	Sports and Upper limb	EL^b^	65 (72.2)	5 (27.8)	<0.001*	142 (86.6)	5 (27.8)	<0.001*
		EM^b^	25 (27.8)	13 (72.2)		22 (13.4)	13 (72.2)	
	Foot and Ankle	EL^b^	2 (40)	1 (10)	0.24	14 (46.7)	1 (10)	0.06
		EM^b^	3 (60)	9 (90)		16 (53.3)	9 (90)	
	Oncology	EL^b^	13 (68.4)	8 (38.1)	0.055	21 (61.8)	8 (38.1)	0.08
		EM^b^	6 (31.6)	13 (61.9)		13 (38.2)	13 (61.9)	
	Pediatrics	EL^b^	52 (77.6)	9 (52.9)	0.06	52 (72.2)	9 (52.9)	0.12
		EM^b^	15 (22.4)	8 (47.1)		20 (27.8)	8 (47.1)	
	Arthroplasty	EL^b^	65 (77.4)	0 (0)	<0.001*	110 (84)	0 (0)	<0.001*
		EM^b^	19 (22.6)	21 (100)		21 (16)	21 (100)	

* Significant p value.

a Quarter.

b Elective surgery (EL) and emergency surgery (EM).

### 3.2 First six months of 2020

We had 700 procedures in the first six months of 2020, of which 416 (59.4%) were elective procedures and 423 (60.4%) were males, with a mean age of 38.85 (Standard deviation = 23.02) years. We increased the number of covering consultants to 17 in this period. All consultants covered on-call, emergency theater, and virtual clinics during the COVID-19 crisis. In the 1st Q, we had 563 procedures with an increase of 24% compared to the 1st Q in 2019. Of those, 175 (31.1%) were emergency surgeries. There were 334 (59.3%) male patients with a mean age of 39.3 (Standard deviation = 22.37) years. While in the 2nd Q, we only performed a total of 137 procedures. Of those, 109 (79.6%) were emergencies. The majority were males 89 (65%), with a mean age of 37.03 (Standard deviation = 25.54) years (Table 1). The specific subspecialty distribution is shown in Table 2.

### 3.3 Effect of COVID-19

**Fig. 2:**
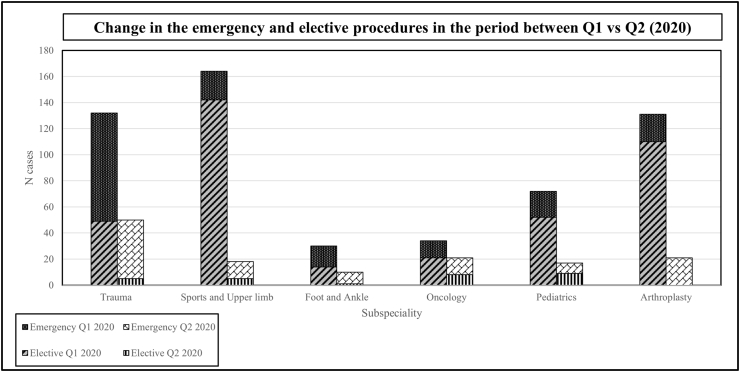
Changes in the emergency and elective procedures in the period between Q1 vs Q2 (2020).

The impact of decisions made in the early COVID-19 phase started to appear by late March through the 2nd Q of 2020. Orthopedic procedures decreased in the 2nd Q of 2020 by 75.6% from the previous quarter of the same year and 61.1% from the 2nd Q in 2019. This significant decrease was mainly because of the decision to hold elective cases, including day surgery and curfew law that started on March 23, 2020 [Figure-1 and Figure-2] [5].

## 4 Discussion

As our fight against COVID-19 crises continues, our way to recovery is not without obstacles, such as variants of the virus. Nationwide and worldwide vaccination programs were created. As of February 19, 2022, 10.53 billion doses have been administered, and around 61.9% of the world population had at least one dose [16]. The previously implemented strategy to fight COVID-19 by curfew and holding elective surgeries nationwide with no specific resumption time has created uncertainty for some patients. Our study measured the effect on orthopedic surgery procedures in a single quarter at the beginning of COVID-19. In our hospital, we successfully increased our orthopedic procedures at the beginning of 2020 (563) by 24% compared to 1st Q 2019 (454) by increasing the number of consultants as well as having more operation time. Unfortunately, this success was interrupted by the COVID-19 pandemic. For the 2nd Q 2020, the number of total procedures decreased by 75.6% as well as 61.1% compared to 1st Q 2020 and 2nd Q 2019, respectively. For emergency procedures, the reduction was around 11.4% for the 2nd Q 2019 and about 37.7% compared to 1st Q 2020. We believe that this considerable difference between 2nd Q and 1st Q emergency procedures is related mainly to the implementation of curfew and the concurrence of 1st Q with the winter season when multiple outdoor-related trauma occurs. This can be seen in the trauma team activity for emergency operations, which decreased by 45.8% from 1st Q 2020 and 18.2% for 2nd Q 2019. More significantly, the number of elective surgeries sustained a severe drop by 92.8% as well as 87.7% compared to 1st Q 2020 and 2nd Q 2019, respectively (Table 1). A study done in the Eastern Provenance of Saudi Arabia on a level-1 trauma center found a 35.4% reduction in orthopedic admissions compared to the year before. The study only counted patients admitted through the emergency room and did not count admissions through the clinic for emergency procedures. The study showed a significant decrease in the length of stay from 18.9 (Standard deviation = 12,7) to 4.3 (Standard deviation = 3.5) days [17]. We believe such a decrease is related to the new policies implemented during the COVID-19 pandemic to decrease exposure to patients, staff and accommodate new admissions in overwhelming circumstances.

Estimating the cancelled procedures is difficult to measure, as well as the financial and social burden on systems, too. Due to a lack of data regarding the actual number of cancelled procedures for patients locally and globally, we designed our study to measure the impact over one quarter (13 weeks) to compare our data with one study that estimated 28,404,603 procedures would be cancelled or delayed worldwide over 12 weeks, representing 72.3% of the total procedures in the same period. They claimed that 90.2% of these cancellations are elective cases; they also concluded that 81.7% of the total elective procedures would be delayed or cancelled compared to 37.7% and 25.4% for cancer as well as obstetric surgeries, respectively. Moreover, they estimated that 82% of orthopedic procedures would be cancelled globally, which was the highest number of cancellations among others (Colorectal, Upper GI and Hepatobiliary, Gynecology, Urology, Plastic surgery, Head and neck) [9]. Furthermore, the best-estimated number of cancelled procedures in Saudi Arabia was 9410 per week, which equals 112,920 for the 12 weeks [9]. Using an online questionnaire answered by 1163 orthopedic surgeons from 85 countries, a worldwide survey found that 848 (73%) have cancelled elective surgeries, and 16% have reduced it by 90%. Additionally, 21% have cancelled all outpatient visits, while 26% have decreased their activity by 90%.

Most orthopedic subspecialties were affected significantly on a global scale by these cancellations. This could be due to the elective nature of most orthopedic procedures. Trauma and oncology units did not have significant restrictions like other subspecialties and were operating as usual [4,[6], [7], [8]]. The oncology chapter of the Saudi orthopedic society issued guidelines that recommended not to delay treatment of any malignant osseous or soft tissue tumors and metastatic fracture or a lesion with impending pathological fracture [18]. In our study, oncology unit cases increased by 10.5% compared to 2nd Q 2019 and decreased by 38.2% from 1st Q 2020. Other subspecialties in our study sustained a decrease compared to 1st Q 2020 by 89% for sports and upper limb, 84% for arthroplasty, and pediatrics by 76.4%. Furthermore, the decrease compared to 2nd Q 2019 was 80% for sports and upper limb, 75% for arthroplasty, and pediatrics by 74.6%. (Table 2). We believe there is a variation in cancellation between countries and hospitals type. We believe there is a variation in cancellation between countries and hospitals type. A study by Arianni et al. assessed hand cases during the COVID-19 crisis in seven countries and encountered a variation in their results regarding injury mechanism, the place where it occurred, and the continuation of elective surgeries in some centers [19].

Cancellations during the early pandemic caused a significant delay for elective procedures and will backlog patients over the post-pandemic phase. An estimation of the recovery would take a median of 45 weeks if surgeries were increased by 20% after recovery for 12 weeks of cancellation only [9]. An expedited plan to overcome such a crisis is essential. Healthcare leaders should guide the community through post-outbreak with the least effect and fastest recovery, which can be very stressful, especially since the economy is a major player in some decisions. From another aspect, COVID-19 has affected not only patients but also orthopedic training programs [20]. This indicates a strong need for planning and implementing a particular curriculum and courses to overcome the defect during this crisis.

Our limitations in our study were the newly developed foot and ankle unit that did not reflect the actual number on their waiting list. Moreover, we could not quantitate the actual waiting list for each subspeciality. Furthermore, we could not calculate each subspeciality's backlog or new bookings. In addition, the COVID-19 crisis extended beyond our study period. And admission status fluctuated in our institute, and once admission resumed, it was closed again due to outbreaks or a surge of cases in the hospital.

## 5 Conclusion

During the COVID-19 crisis, elective surgeries were held in our hospital and most of Saudi Arabia. Our cohort showed a significant decrease during this period. All subspecialties were affected by holding elective procedures, but some were affected more because of the elective nature of their operations, such as sports and upper limb, pediatric, and arthroplasty. We believe that we need further research and better evaluation to find a plan to overcome this significant backlog of patients, especially those who are disparately in need of intervention to restore a function or alleviate their pain.

## Ethical approval

Prior to our data collection, the research ethics committee approval #1450 was obtained from Prince Sultan Military Medical City on the 14th of December 2020.

## Sources of funding

This study did not receive any specific grant from funding agencies in the public, commercial, or not-for-profit sectors.

## Author contribution

Abdulellah S. Abunayan, contributed to the study idea, design, data analysis, manuscript writing and supervised data collection. Bader M. Aljadaan, revised the ethics committee draft and the final manuscript. Mohammed A. Almudayfir, Sayaf H. Alshareef, and Abdulaziz F. Alamer; participated in data collection, entry, organization, and coding. All authors have critically revised and approved the final manuscript.

## Registration of research studies

Name of the registry: Research Registry.

Unique Identifying number or registration ID: researchregistry8037.

Hyperlink to your specific registration (must be publicly accessible and will be checked): https://www.researchregistry.com/register-now#home/registrationdetails/62b5c54e239f9a001edd60fc/

## Guarantor

Dr. Abdulellah S. Abunayan, Primary and corresponding author of the study. Orthopedic resident at Prince Sultan Military Medical City, Riyadh 11451, Kingdom of Saudi Arabia. Phone: +966556224488, Email: abdulellah.abunayan@gmail.com.

## Provenance and peer review

Not commissioned, externally peer reviewed.

## Consent

No informed consent needed in our study design.

## Declaration of competing interest

No conflict of interest to disclose.
